# De novo transcriptome analysis of petal senescence in *Gardenia jasminoides* Ellis

**DOI:** 10.1186/1471-2164-15-554

**Published:** 2014-07-04

**Authors:** Georgios F Tsanakas, Maria E Manioudaki, Athanasios S Economou, Panagiotis Kalaitzis

**Affiliations:** Department of Horticultural Genetics & Biotechnology, Mediterranean Agronomic Institute of Chania (MAICh), Crete, Greece; School of Agriculture, Aristotle University of Thessaloniki, Thessaloniki, Greece; Mediterranean Agronomic Institute of Chania (MAICh), Alsyllio Agrokepio, 1 Makedonias str, PO Box 85 Chania, 73100 Crete Greece

**Keywords:** Gardenia, Transcriptome, SSR markers, Transcription factors, Petal senescence

## Abstract

**Background:**

The petal senescence of ethylene insensitive species has not been investigated thoroughly while little is known about the temporal and tissue specific expression patterns of transcription factors (TFs) in this developmental process. Even less is known on flower senescence of the ornamental pot plant *Gardenia jasminoides*, a non climacteric flower with significant commercial value.

**Results:**

We initiated a *de novo* transcriptome study to investigate the petal senescence in four developmental stages of cut gardenia flowers considering that the visible symptoms of senescence appear within 4 days of flower opening. *De novo* assembly of transcriptome sequencing resulted in 102,263 contigs with mean length of 360 nucleotides that generated 57,503 unigenes. These were further clustered into 20,970 clusters and 36,533 singletons. The comparison of the consecutive developmental stages resulted in 180 common, differentially expressed unigenes. A large number of Simple Sequence Repeats were also identified comprising a large number of dinucleotides and trinucleotides. The prevailing families of differentially expressed TFs comprise the AP2/EREBP, WRKY and the bHLH. There are 81 differentially expressed TFs when the symptoms of flower senescence become visible with the most prevailing being the WRKY family with 19 unigenes. No other WRKY TFs had been identified up to now in petal senescence of ethylene insensitive species. A large number of differentially expressed genes were identified at the initiation of visible symptoms of senescence compared to the open flower stage indicating a significant shift in the expression profiles which might be coordinated by up-regulated and/or down-regulated TFs. The expression of 16 genes that belong to the TF families of WRKY, bHLH and the ethylene sensing pathway was validated using qRT – PCR.

**Conclusion:**

This *de novo* transcriptome analysis resulted in the identification of TFs with specific temporal expression patterns such as two WRKYs and one bHLH, which might play the role of senescence progression regulators. Further research is required to investigate their role in gardenia flowers in order to develop tools to delay petal senescence.

**Electronic supplementary material:**

The online version of this article (doi:10.1186/1471-2164-15-554) contains supplementary material, which is available to authorized users.

## Background

One of the main issues that floriculture industry has to confront is flower senescence, a term that signifies all those processes that follow physiological maturity and lead to the death of a whole plant, tissue or cell and represents the last stage of flower development. A series of events take place during flower senescence resulting in highly regulated, genetically programmed and developmentally controlled morphological, physiological and biochemical changes. By the end of this process, the flower petals wilt, lose colour and abscise [1].

Plants are classified as climacteric or non-climacteric, depending on their ethylene production rate. Climacteric flowers such as carnation and petunia are characterized by the climacteric ethylene and respiration rate production that promotes senescence, while treatment with exogenous ethylene results in acceleration of senescence [2]. In addition, an inhibitor of prolyl 4 hydroxylase activity, pyridine 2,4 dicarboxylate, suppressed the climacteric ethylene production in cut carnation flowers [3]. In contrast to carnation and petunia, the flowers of lilies (Lilium spp.), gladiolus, tulip (Tulipa spp.), iris and morning glory (Ipomoea nil) are classified as non-climacteric since they are not responsive to ethylene and exogenous application has little or no effect on petal senescence [4]. Ethylene acts as a trigger to flower senescence in long-lived flowers, possibly as a mechanism to terminate flower life after pollination. In short-lived flowers on the contrary, such a mechanism would not be beneficial since the life of individual flowers is very short [1].

Genes that are associated with ethylene sensing and sensitivity have also been found in ethylene insensitive plants such as the two homologues of Arabidopsis ethylene receptor ERS1 which were found in gladiolus [5]. Over the last years several signalling components have been found to be associated with petal senescence in climacteric species, such as the F-box proteins EBF1/EBF2 that recognize and degrade EIN3 through the ubiquitin/26S proteasome pathway [6, 7]. They also interact with EIN2 to function in the modulation of ethylene signalling [8] as well as with EIN3, EIL1 and ASK1 which is a component of the SCF ubiquitin ligase complex.

In Arabidopsis petals, 316 TFs showed differential expression patterns of which 130 changed expressions only in petal but not in leaf or silique senescence [9]. These TFs are members of 47 gene families while the three most represented families with up-regulated patterns of expression were the ERF, NAC and WRKY [9].

The EIN/EIL transcription factors (TFs) are targeting genes with an Ethylene Responsive Element in their promoter. They belong to the AP2/EREBP-type TFs and are correlated with stress response [10–12], ripening and senescence [13, 14]. They are also correlated with the effect of sucrose in delaying senescence [15].

In addition to ERFs there are other families of TFs associated with senescence such as the WRKYs and basic helix loop helix (bHLH). The WRKY TF family constitutes a large family with at least one WRKY domain of approximately 60 amino acids. They are unique to the plant kingdom and some of its members have a very important role in the regulation of leaf senescence, response of the plant to bacterial infection, signaling pathways and many biotic and abiotic stresses [16–18]. WRKY target genes with a W-box (TTGAC or TTGACC/T) in their promoter [19, 20] regulating, among others, the expression of senescence associated with related genes [16, 21]. Members of the WRKY family might constitute a hub transcription factor during senescence via mediation of jasmonic acid and salicylic acid signaling [22, 23].

The bHLH proteins can be found both in mammals and plants [24] and are known to interact with other proteins and transcription factors such as MYB [25, 26]. They were also associated with a series of developmental phenomena in the plant, such as the development of root hair and leaf trichomes [27, 28], cell proliferation and cell differentiation [24, 27].

However, little is known about the expression of TFs in petal senescence of ethylene insensitive species. In alstroemeria 21 TFs were identified among 2000 ESTs [29], while other TFs were identified in daffodil [30] and iris [31], while in morning glory (Ipomoea nil) suppression of a leucine-rich repeat transmembrane protein kinase displayed accelerated petal senescence [4].

Recently, there is an increased interest for the ornamental pot plant *Gardenia jasminoides* Ellis, since apart from its wide use as a floral plant, it is of high pharmaceutical value considering that contains the substance geniposide that can be transformed into the anti-inflammatory and anti-angiogenesis agent genipin [32–34]. The physical map of gardenia is not available yet, while molecular studies are restricted to discriminate gardenia species for systematic reasons or to study the phytogeography of the wild and commercial populations [35–40]. Gardenia is considered a non-climacteric species with flowers that do not produce detectable levels of ethylene therefore is not included in the list of climacteric flower crops [2]. We initiated a study to investigate the petal senescence of cut gardenia flowers at the transcriptome level considering that the visible symptoms of senescence appear within four days. Towards this direction we used Next Generation Sequencing Technologies, in particular Illumina HiSeqTM 2000 for de novo sequencing and characterization of gardenia petals transcriptome at four stages of senescence progression.

## Results

**Figure 1:**
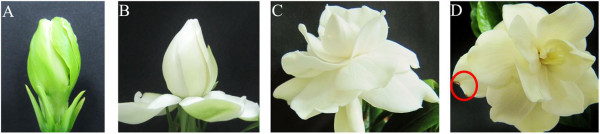
**Blooming stages of gardenia flower. (A)** Flower bud, one day prior to opening **(B)** Opened flower with horizontally aligned outer petals **(C)** Fully opened flower with expanded petals, **(D)** Senescent flower that has been discolored to yellow pale and the first necrotic spot in petals are visible.

The senescence progress of gardenia flowers is completed within four days, therefore it was divided in four developmental stages. The first stage (A) comprises closed buds ready to open and the second stage (B) open flowers with the outer whorl of petals at a 90° angle to the flower stalk. At the third developmental stage (C) the flowers are fully open, while at the fourth stage (D) the yellow discoloration of petals was initiated as well as the appearance of brown spots (Figure 1). Total RNA was extracted from each development stage and used for de novo transcriptome sequencing with Illumina HiSeqTM 2000.

### cDNA sequence generation, de novo assembly and quantification of gene expression

Sequencing of the gardenia transcriptome resulted in a total of 50,335,672 reads that were obtained after cleaning the raw data (Additional file 1). De novo assembly of these reads resulted in 102,263 contigs with mean length of 360 nt that generated 57,503 unigenes with a mean length of 796 nt. These were further clustered into 20,970 clusters and 36,533 singletons. Quantification of gene expression for the four developmental stages of gardenia flower senescence, the uniquely mapped reads for each stage and the corresponding unigenes are shown in Additional file 2. The E-value distribution of the top hits in NCBI non-redundant (Nr) database, the similarity distribution as well as the species distribution are included in Additional file 3.

### Gene Ontology annotation of gardenia transcriptome

**Figure 2:**
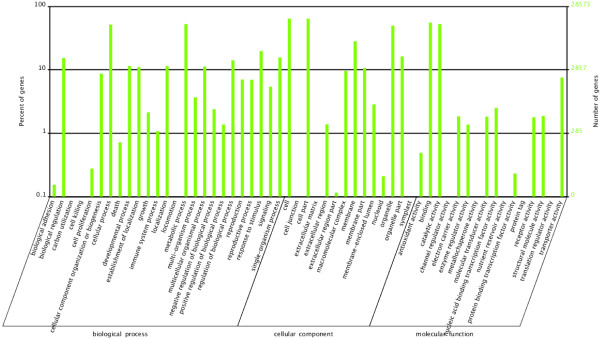
**Gene Ontology classification of the total assembled unigenes.** The left side and the right side of the panel show the percentage of genes and the number of genes that are classified in the corresponding term, respectively.

The gardenia genes were classified according to the Gene Ontology annotation in three categories, namely Biological Process, Cellular Component and Molecular Function (Figure 2).

In the category “Biological Process” most genes were associated with “biological regulation”, “cellular process”, “metabolic process”, “regulation of biological process”, “response to stimulus”, “single-organism process” and “signaling”. For the category “Cellular Component”, the dominant subcategories were those genes associated with “cell” and “cell part”, “membrane”, “organelle” and “organelle part”. In the category “Molecular Function”, “binding” and “catalytic activity” were the prevailing terms. Overall, in the present study, genes that belong to the categories “cellular process”, “metabolic process”, “response to stimulus” (biological process), “cell”, “cell part”, “organelle” (cellular component) and “binding”, “catalytic activity”, “metabolic activity” (molecular function) were the most highly represented. The above mentioned gene categories were the most highly represented in other transcriptome studies as well, such as in safflower and in chrysanthemum [41, 42].

### Clusters of Orthologus Groups (COG) annotation

**Figure 3:**
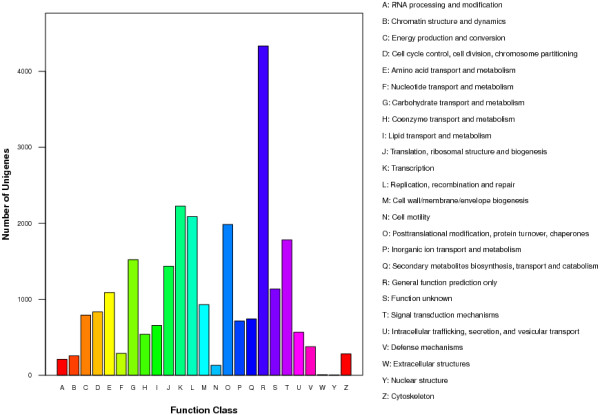
**COG classification of the gardenia transcriptome.**

Search against the COG database resulted in the classification of 13,462 unigenes in 24 COG categories. As it can be seen in Figure 3, the “General Function prediction only” represents the largest group with 4,333 unigenes followed by “Transcription” (2,227 unigenes), “Replication, recombination and repair” (2,089 unigenes), “Posttranslational modification, protein turnover, chaperones” (1,984 unigenes) and “Signal transduction mechanisms” (1,782 unigenes).

### Differentially expressed unigenes

**Figure 4:**
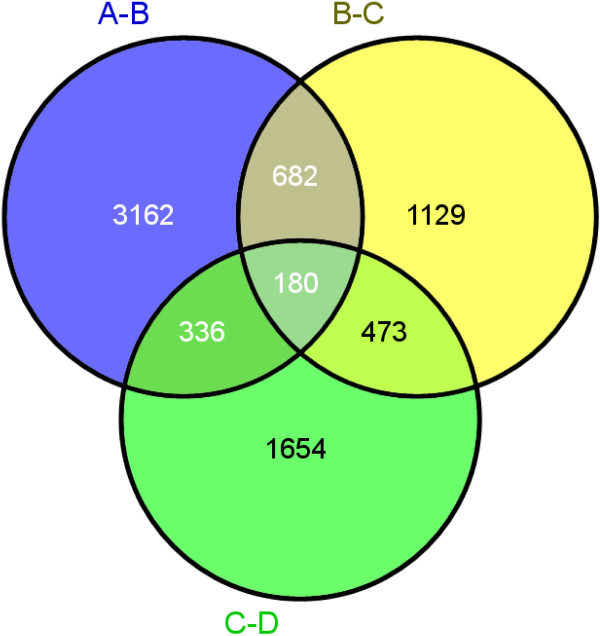
**Venn diagrams of the unique, differentially expressed unigenes in three pairs of the developmental stages. (A-B)** Differentially expressed unigenes in the transition from stage **A** to stage **B (B-C)** Differentially expressed unigenes in the transition from stage **B** to stage **C (C-D)** Differentially expressed unigenes in the transition from stage **C** to stage **D**.

The unigenes that are differentially expressed in pairs of two consecutive stages of senescence progress are shown in Table 1. As the gardenia flower develops from closed bud of stage A to early open flower of stage B, 4,360 unigenes are differentially expressed, of which 3,402 are down-regulated and 958 are up-regulated (Table 1). Among them, there are 3,162 unique unigenes which are differentially expressed only in these two developmental stages but not in the other consecutive stages (Figure 4). The progression of the partially open into a fully open flower at stage C is characterized by 2,464 differentially expressed unigenes of which 1,654 are down-regulated and 810 are up-regulated (Table 1). Among them, there are 1,129 unique, differentially expressed transcripts (Figure 4). The developmental stage D with visible symptoms of senescence in comparison to stage C is characterized by 2,643 differentially expressed unigenes of which 1,061 are down-regulated and 1,582 are up-regulated (Table 1). Among them, there are 1,654 unique, differentially expressed transcripts (Figure 4).

**Table 1:** **Up- and down-regulated transcripts during the progression of senescence**

	A-B	B-C	C-D
↓	3,402	1,654	1,061
↑	958	810	1,582
Total	4,360	2,464	2,643

Moreover, 682 differentially expressed transcripts are common between the stages A and B and those of B and C (Figure 4). Furthermore, 473 differentially expressed transcripts are found in stages B and C, and C and D, while 336 differentially expressed transcripts are common in the stages A and B, and C and D. The comparison of the consecutive developmental stages resulted in 180 common, differentially expressed unigenes. Among them, 21 unigenes were up-regulated, while 66 were down-regulated in all four developmental stages.

As petal senescence progresses, the number of down-regulated unigenes decreases, while that of up-regulated increases only at the stage D of visible symptoms of senescence (Table 1).

The comparison between stage A and the other developmental stages B, C and D indicates that as senescence progresses, the number of differentially expressed unigenes increases (Table 2). Specifically, 4,360, 7,658 and 10,401 unigenes are differentially expressed between stages A and B, C and D, respectively, indicating significant variation in gene expression profiles among developmental stages.

**Figure 5:**
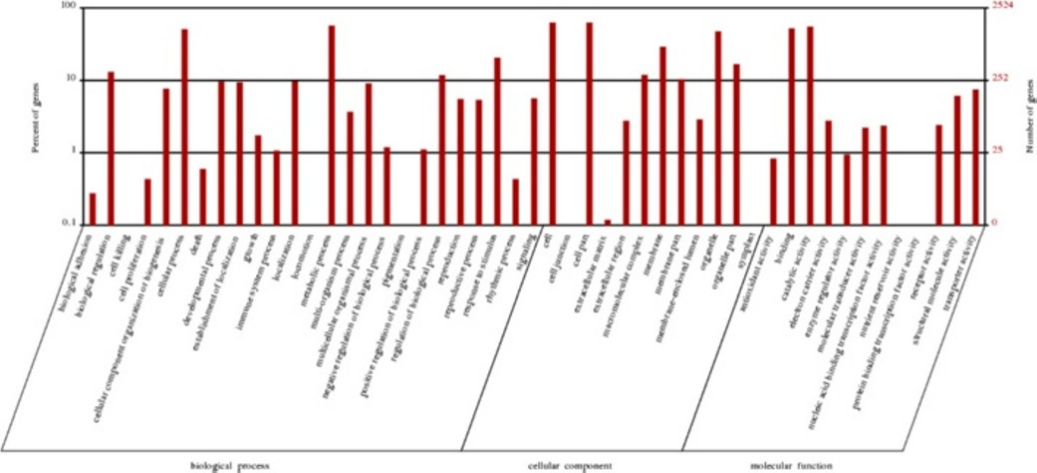
**Gene Ontology classification of the differentially expressed unigenes of the progression from stage A to stage B.** The left side and the right side of the panel show the percentage of genes and the number of genes that are classified in the corresponding term, respectively.

**Figure 6:**
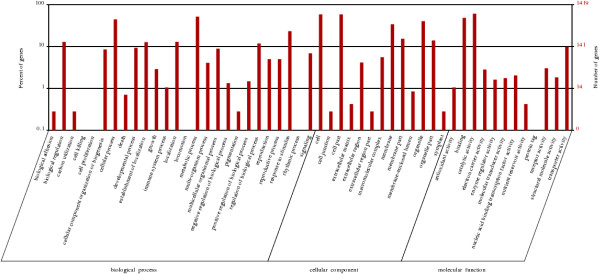
**Gene Ontology classification of the differentially expressed unigenes of the progression from stage B to stage C.** The left side and the right side of the panel show the percentage of genes and the number of genes that are classified in the corresponding term, respectively.

**Figure 7:**
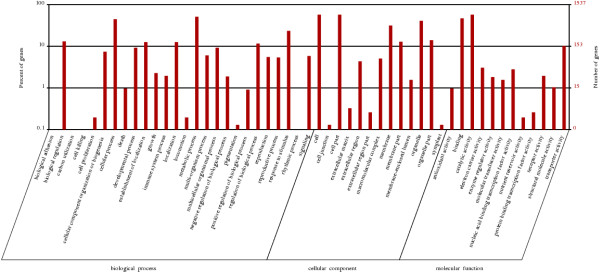
**Gene Ontology classification of the differentially expressed unigenes of the progression from stage C to stage D.** The left side and the right side of the panel show the percentage of genes and the number of genes that are classified in the corresponding term, respectively.

The functional classification of the differentially expressed unigenes in the four developmental stages was performed as shown in Figures 5, 6 and 7.

**Table 2:** **Differentially expressed transcripts**

A-B	A-C	A-D
4,360	7,658	10,401

### Identification of simple sequence repeats

A total of 9,549 SSRs were indentified in 7,641 (13.3%) transcripts of gardenia (Additional file 4). Among them, 1,398 (18.3%) transcripts contained more than one SSR, while the largest category of the SSRs was mononucleotides (3129, 32.7%), followed by trinucleotides (2,937, 30.7%) and dinucleotides (2,853, 29.8%). The A/T (30.9%) accounted for the 94.5% of the mononucleotide repeats, the AG/CT (19.2%) accounted for the 69.5% of dinucleotides, while the AAG/CTT (8%), AGG/CCT (7.7%) and AAC/GGT (3.5%) accounted for the 62.4% of the trinucleotides. A small number of tetranucleotides (230, 2.4%), pentanucleotides (209, 2.2%) and hexanucleotide (190, 2.0%) were also identified.

### Differentially expressed transcription factors during petal senescence

**Figure 8:**
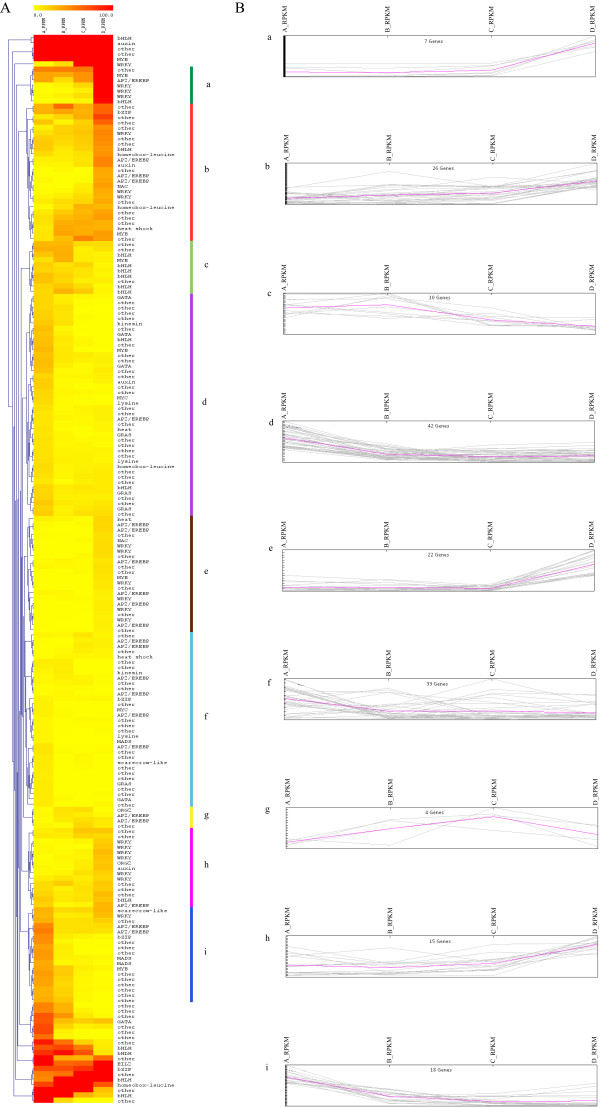
**Hierarchical clustering and expression patterns of differentially expressed TFs during senescence. (A)** Hierarchical clustering of the differentially expressed TFs using Euclidean distance. On the right side bars of various colours are used to determine distinct clusters **(B)** Expression patterns that correspond to the clusters of the histogram. Each line represents a transcript in the corresponding senescent stage where expression values are represented as RPKM values.

**Figure 9:**
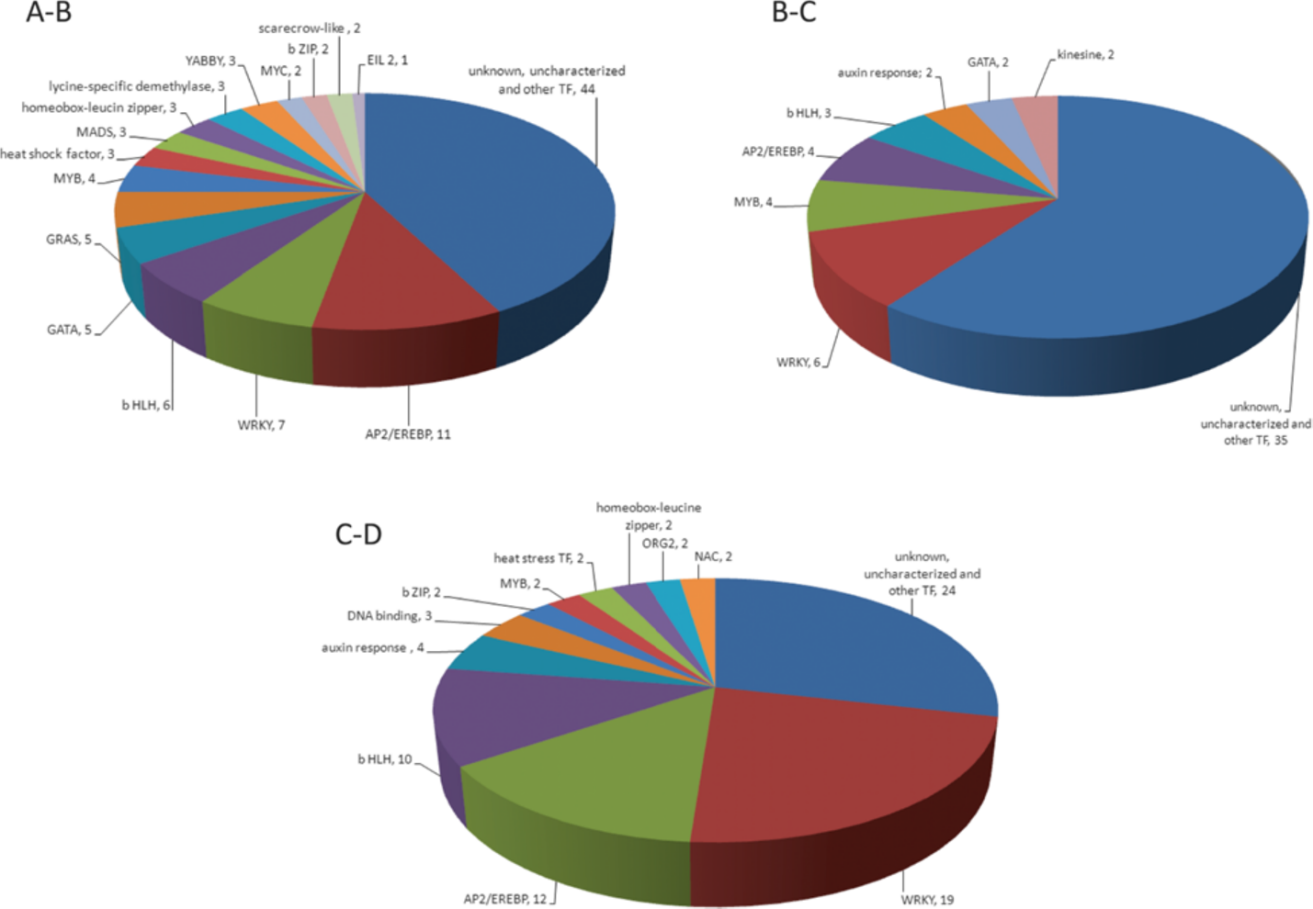
**The differentially expressed TF unigenes between sequential stages of senescence.**

The 202 differentially expressed unigenes that encode TFs within the four developmental stages are shown in Figure 8. Among them, there are 107 TFs with differential expression patterns during progression to stage B. The prevailing family of transcription factors is the “AP2/EREBP” family with 11 unigenes, followed by the “WRKY” transcription factor family with 7 unigenes and the “bHLH” TFs with 6 unigenes, while there are 44 unknown/uncharacterized TFs (Figure 9A-B). Additional differentially expressed TFs include the “GATA” and “GRAS” with 5 unigenes, the “MYB” family with 4 unigenes and the “heat shock / stress”, “MADS”, “homeobox-leucine zipper”, “lycine-specific dimethylase” and “YABBI” families with 3 unigenes.

As senescence progresses from partial to fully open flower, 58 TFs are differentially expressed comprising the “WRKY” family with 6 unigenes, followed by the “MYB” and the “AP2/EREBP” family with 4, the “bHLH” TFs with 3, and the “auxin response”, “GATA” and “kinesine” with 2 unigenes (Figure 9B-C).

There are 81 differentially expressed TFs when the symptoms of flower senescence become visible at stage D. The most prevailing TF families include the “WRKY” family with 19 unigenes, the “bHLH” with 10, the “AP2/EREBP” with 12 while “auxin response” and “DNA binding” comprise 4 and 3 unigenes, respectively (Figure 9C-D).The clusters a, b, e and h mainly comprise WRKY TFs and their expression levels are higher in stage D while the cluster c mainly comprise bHLH TFs and their transcript abundance decreases after stage B (Figure 8). The GRAS and GATA TFs are grouped in cluster d and their expression decreases during senescence progression. AP2/EREBP TFs are mainly present in five clusters, b, e, f, g and i showing different patterns of expression. In clusters b and e the expression of the unigenes is higher in stage D, in clusters f and g is higher in the intermediate stages B or C and in cluster i the expression of the unigenes is high at the first stage and then decreases throughout senescence (Figure 8).

### Kyoto Encyclopedia of Genes and Genomes (KEGG) pathway mapping

The 21,614 unigenes were assigned to 128 KEGG pathways. The most abundant pathways were ‘Metabolic pathways’ (5,001), ‘Biosynthesis of secondary metabolites’ (2,497), ‘Plant-pathogen interaction’ (1,146), ‘Plant hormone signal transduction’ (990), ‘Spliceosome’ (750), ‘RNA transport’ (676), ‘Endocytosis’ (603) and ‘Starch and sucrose metabolism’ (578). The prevailing categories, based on transcript abundance, between stages A and B were ‘Metabolic pathways’, ‘Biosynthesis of secondary metabolites’, ‘Ribosome’ and ‘Plant-pathogen interaction’, while between stages B and C were ‘Metabolic pathways’, ‘Biosynthesis of secondary metabolites’, ‘Starch and sucrose metabolism’, ‘Plant hormone signal transduction’ and ‘Plant-pathogen interaction’. The prevailing categories between stages C and D included ‘Metabolic pathways’, ‘Biosynthesis of secondary metabolites’, ‘Plant-pathogen interaction’, ‘Plant hormone signal transduction’ and ‘Starch and sucrose metabolism’, while between the stages A and D included ‘Metabolic pathways’, ‘Biosynthesis of secondary metabolites’, ‘Plant-pathogen interaction’, ‘Plant hormone signal transduction’ and ‘Ribosome’.

**Figure 10:**
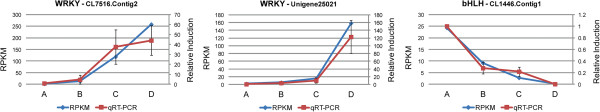
**Differentially expressed TFs throughout senescence.** Patterns of expression of two WRKY transcripts, the CL7516.Contig2 and the Unigene25021 and one bHLH transcript, the CL1446.Contig1 determined by qRT-PCR (relative induction compared to stage A) and RNAseq (RPKM values). Error bars represent the standard deviation of the means.

There are 19 WRKY unigenes which are differentially expressed when the visible symptoms of senescence appeared, while 13 among them are unique to this developmental stage indicating specific involvement on the initiation of senescence symptoms. Only two WRKY and one bHLH unigenes showed a progressive increase and decrease, respectively in expression throughout petal senescence (Figure 10).

### Ethylene sensing in the non-climacteric flower of gardenia

**Figure 11:**
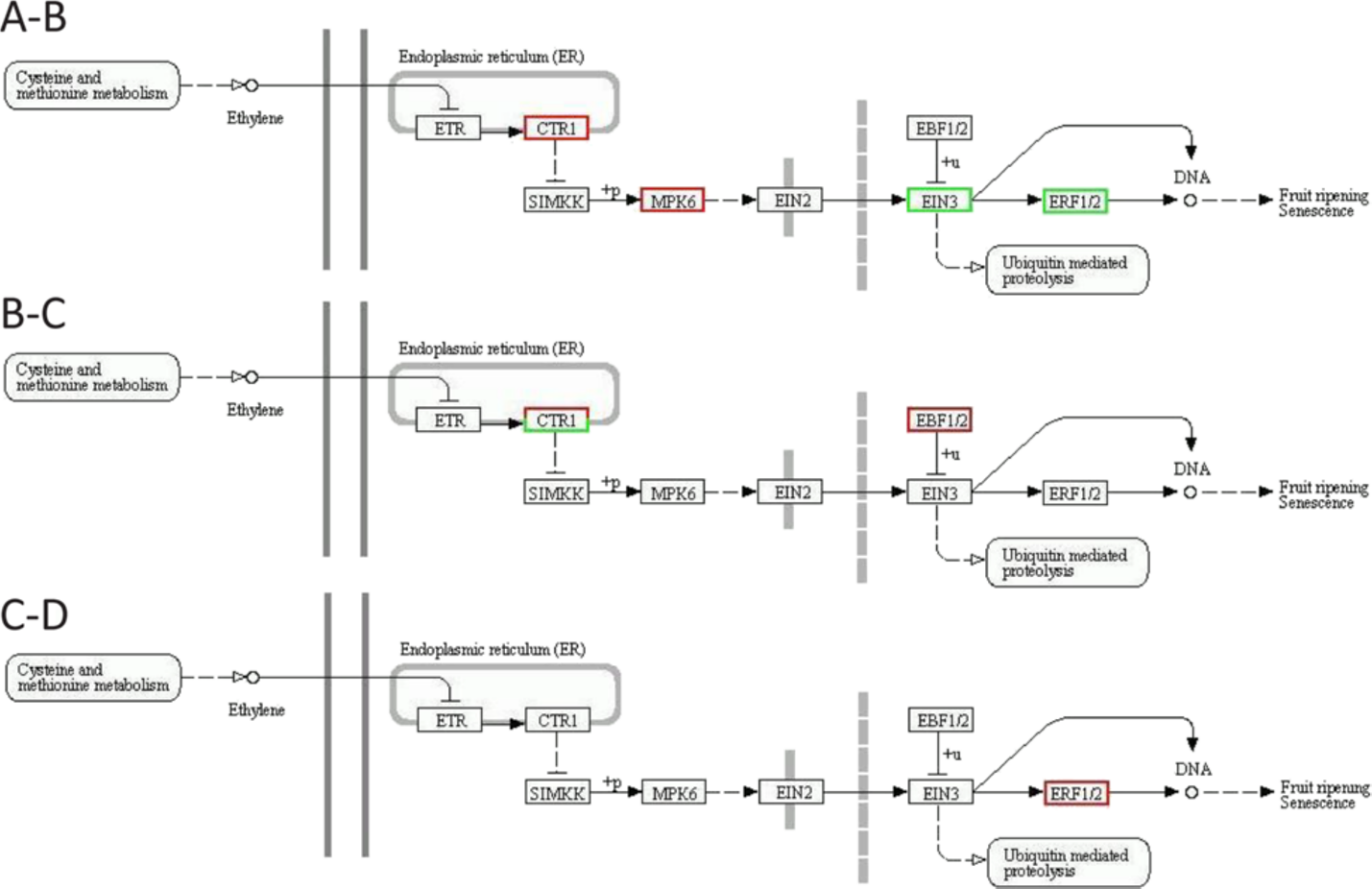
**The ethylene sensing pathway. (A-B)** Unigenes that participate in the ethylene sensing pathway and are differentially expressed in the transition from stage A to stage B **(B-C)** Unigenes that participate in the ethylene sensing pathway and are differentially expressed in the transition from stage B to stage C **(C-D)** Unigenes that participate in the ethylene sensing pathway and are differentially expressed in the transition from stage C to stage D. A red frame indicates up-regulated unigenes and a green frame indicates down-regulated unigenes.

Differentially expressed unigenes were assigned to KEGG pathways associated with ethylene sensing (Figure 11). The CTR1 and the MPK6 unigenes are up-regulated, while the EIN3 and the ERF1/ERF2 are down-regulated during the transition from the stage A of closed buds ready to open to stage B of open flowers with the outer whorl of petals at a 90° angle to the flower stalk (Figure 11). As senescence progresses to fully open flowers, two CTR1 unigenes are still up-regulated, while one is-down regulated. In addition, two EBF1/EBF2 unigenes are up-regulated (Figure 11). When visible symptoms of senescence appear in the petals, four ERF1/ERF2 unigenes were up-regulated (Figure 11). These results indicate progressive up-regulation of ethylene sensing components during senescence development.

### Validation of expression of selected unigenes using real time PCR

**Figure 12:**
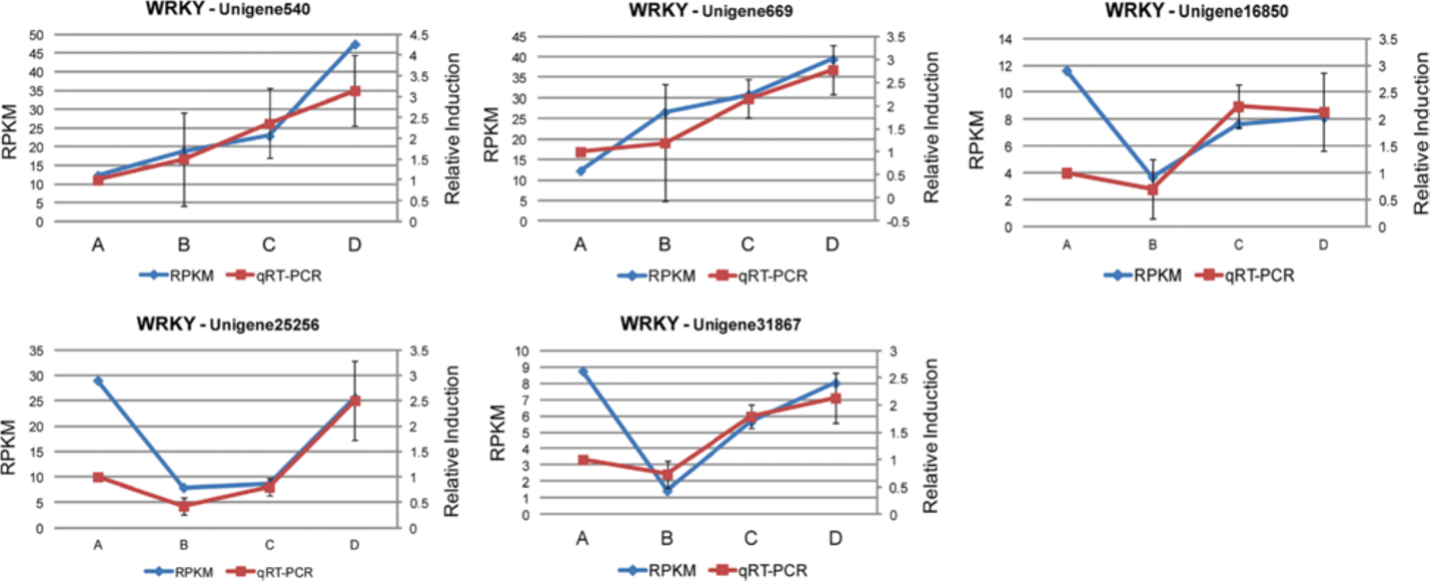
**Expression profiles of five WRKY transcripts.** Patterns of expression of five WRKY transcripts, the Unigene540, Unigene669, Unigene16850, Unigene25256 and Unigene31867 determined by qRT-PCR (relative induction compared to stage A) and RNAseq analysis (RPKM values). Error bars represent the standard deviation of the means.

**Figure 13:**
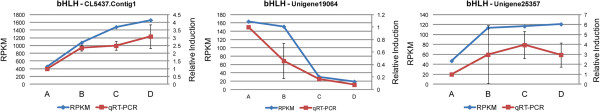
**Expression profiles of three bHLH transcripts.** Patterns of expression of three bHLH transcripts, the CL5437.Contig1, Unigene19064 and Unigene25357 determined by qRT-PCR (relative induction compared to stage A) and RNAseq analysis (RPKM values). Error bars represent the standard deviation of the means.

**Figure 14:**
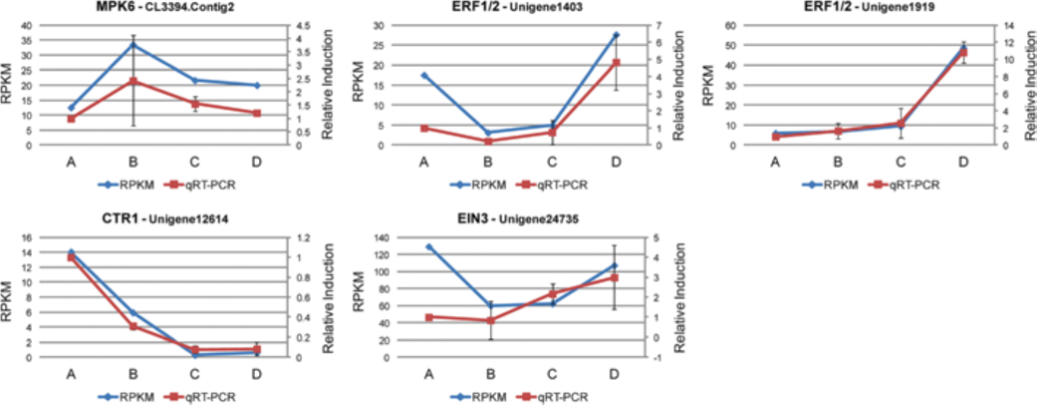
**Expression profiles of five ethylene sensing transcripts.** Patterns of expression of MPK6 (CL3394.Contig2), ERF1/2 (Unigene1403), ERF1/2 (Unigene1919), CTR1 (Unigene12614) and EIN3 (Unigene24735), determined by qRT-PCR (relative induction compared to stage A) and RNAseq analysis (RPKM values). Error bars represent the standard deviation of the means.

The patterns of expression of selected unigenes during the four stages of petal senescence were determined by real-time PCR using two reference genes, actin (CL715.Contig1) and PP2A (Protein Phosphate 2A) (Unigene23262). Two WRKY and one bHLH unigenes were up- and down-regulated, respectively throughout senescence showing similar to RNAseq patterns of expression (Figure 10). In addition, the transcript abundance of five differentially expressed WRKYs (Figure 12) and three differentially expressed bHLHs (Figure 13) were also determined during senescence. Their patterns of expression are similar to those determined using RNAseq indicating that quantification of expression profiles with this technological platform might be considered reliable. Moreover, five ethylene sensing components such as CTR1, MPK6, EIN3 and two ERF1/2 were further characterized by quantifying their mRNA levels during petal senescence with real-time PCR (Figure 14). Each one of the five ethylene signal transduction pathway components showed a distinct pattern of expression confirming the RNAseq analysis (Figure 14).

## Discussion

A large scale transcriptome analysis of petal senescence in a non-climacteric species, *Gardenia jasminoides* Ellis, was performed. Four distinct developmental stages of senescence were analyzed leading to the identification of 57,503 unigenes which were annotated against known sequences in various databases, whereas differentially expressed unigenes were also identified. The relative transcript levels of 16 differentially expressed unigenes were further validated by using real time PCR.

Annotated unigenes in all databases (NR, NT, Swiss-Prot, KEGG, COG, and GO) comprised 68.6% of the total number of unigenes, which should be considered adequate taking into account the lack of sequencing data on gardenia species. In other similar studies in other species, such as bamboo and cucumber, the annotated unigenes represented 55% and 72% of the total number of unigenes, respectively [43, 44]. The E-value distribution of the top hits in NR database also showed high homology, even in the absence of a reference genome.

The functional classification of gardenia genes according to GO annotation identified genes associated to ‘Biological Process’, ‘Cellular Component’ and ‘Molecular Function’ categories. Genes annotated to ‘Biological process’ were identified during flower development of Eustoma grandiflorum [45] and in three genotypes of *Eichornia paniculata* [46], while those involved in metabolic process were also identified in carnation, snapdragon, alstroemeria, tobacco and Arabidopsis [9, 29, 47–49] indicating significant metabolic activity during petal senescence progression.

Gene categories associated with ‘Cellular Component’ were also identified during flower senescence of bamboo [43] implicating the “membrane” associated genes to alterations in membrane permeability due to changes in composition and structure of the lipid bilayers [50]. Gene categories associated with ‘Molecular Function’ were also found to be highly represented during flower formation and petal senescence in safflower, carnation, orchids and wallflower [41, 51–53]. Search against the COG database that briefly shows the proteomic profile indicated similarities between gardenia petal senescence and *Dendrocalamus latiflorus* flower development [43].

Microsatellites or Simple Sequence Repeats (SSRs) are considered reliable and informative markers in plant research. In this study a total number of 9,549 SSRs were identified in 7,641 sequences. The majority of SSRs were mononucleotides followed by trinucleotides and dinucleotide repeats, as is the case in chickpea [54]. These SSRs can be used for fingerprinting and breeding efforts within gardenia species.

A large number of genes are differentially expressed among the four different developmental stages of senescence. Among them, a plethora of transcription factors such as the WRKY, AP2/EREBP and bHLH were the most highly represented.

The WRKY family comprises the highest number (19) of differentially expressed unigenes in the final stage of gardenia petal senescence indicating significant involvement in this process compared to the other TF families. No other WRKY TFs had been identified up to now in petal senescence of ethylene insensitive species [2]. It is of particular interest the fact that there are 1,582 differentially expressed genes at the initiation of visible symptoms of senescence compared to the open flower stage indicating a significant shift in the expression profiles, which might be coordinated by up-regulated TFs in these two developmental stages. Such candidate TFs might be the two WRKYs (‘CL7516.Contig2’ and ‘Unigene25021’) exhibiting expression profiles of up-regulation throughout petal senescence. These patterns were also confirmed using real time PCR (Figure 10). Moreover, another five WRKY TFs were shown to be up-regulated mainly at the last stage of senescence progression according to real time PCR analysis. The WRKY TFs are well known to be up-regulated during senescence in various organs in Arabidopsis, while petal and silique senescence was associated with the expression of more members of this gene family [9]. In addition, WRKY TFs were also found to be highly abundant during bamboo flower development [43].

In gardenia petals, the bHLH unigenes are expressed in a stage specific manner and the expression pattern is distinct related to the other TFs while only one unigene (‘CL1446.Contig1’) is differentially expressed throughout senescence. The expression of this unigene is progressively down-regulated according to RNAseq and real-time PCR analysis (Figure 10). Moreover, there are ten differentially expressed unigenes at the stage of visible symptoms of senescence appearance; two were up- and eight were down-regulated (Table 3). Real time PCR analysis confirmed the expression profiles of three of them; two showed an increase in transcript abundance and one a decrease (Figure 13). The bHLH TFs are known to be associated with flower organogenesis, floral development and promotion of the proliferation inside the flower on a tissue-specific way [55–57]. They are also known to interact with other proteins and TFs such as the MYB families [25, 26] which are also differentially expressed throughout gardenia flower senescence (Figure 8).

**Table 3:** **Common and unique WRKY and bHLH unigenes across the stage comparisons**

	Α-Β	Β-C	C-D
**WRKY**			
**bHLH**			

The spots represent differentially expressed unigenes of the transcription factors WRKY and bHLH during the progress of senescence. Each spot represents a single unigene while spots with the same colour refer to the same unigene.

Although gardenia is a non-climacteric flower, 12 AP2/EREBP TF unigenes were found to be differentially expressed with 11 of them up- and one down-regulated when the visible symptoms of senescence appeared. In addition to these positive TF regulators of ethylene response, other ethylene signaling components seem to be up-regulated in earlier stages of flower senescence such as the CTR1 and EBF1/EBF2 unigenes, which serve as negative regulators of ethylene response. These results suggest a pattern of expression for ethylene sensing and response genes in which the negative regulators are expressed early in flower senescence, while the positive regulators at later stages in order to possibly accelerate this developmental program.

The expression patterns of these five ethylene sensing components were validated using real time PCR suggesting involvement of ethylene in gardenia petal senescence.

Overall, the mRNA levels of several genes of interest by RNAseq analysis were further validated by real time PCR analysis indicating the reliability of RNAseq data.

The transcriptome analysis of gardenia petal senescence resulted in the identification of several TFs which might serve as targets for manipulation of their expression using genetic engineering approaches.

## Conclusion

The de novo transcriptome analysis of *Gardenia jasminoides* Ellis resulted in the identification and functional classification of differentially expressed unigenes during petal senescence as well as in a large number of SSR markers. The assignment of these unigenes in KEGG pathways revealed potential involvement of ethylene sensing components in this developmental program. Moreover, differentially expressed transcription factors such as two WRKYs and one bHLH were identified showing specific temporal expression patterns which might play the role of senescence progression regulators. Their expression patterns as well as of other members of these gene families were further validated using real time PCR approaches. However, further research is required to investigate the specific role of transcription factors in gardenia flowers in order to develop molecular tools for delaying petal senescence.

## Methods

### Plant material

Thirty five pot-plants of *Gardenia jasminoides* Ellis were cultivated in the greenhouse of the Laboratory of Floriculture at the Aristotle University of Thessaloniki, Greece. All plants were propagated by terminal shoot cuttings (7-8 cm in length), from a single mother plant that was provided by the cooperative “Gardenia Growers Group” of Magnesia, Greece. The basal portion (1 cm) of each cutting was dipped into 500 ppm of K-IBA solution and then planted in 2:1 peat:perlite substrate, in a fog propagation system (RH 95-98%). Two months later, the rooted cuttings were transplanted in 1,3 L pots filled with peat and grown identically in the greenhouse for one year, according to the commercial cultivation techniques.

### Floral stages, RNA extraction and Illumina HiSeqTM 2000

Twenty five buds or flowers per stage were randomly selected from the above plants and harvested. Since the senescence progression of gardenia flowers is completed within four days, we divided the phenomenon in four developmental stages. The first stage (A) comprises closed buds ready to open and the second stage (B) open flowers with the outer whorl of petals at a 90° angle to the flower stalk. At the third developmental stage (C) the flowers are fully open, while at the fourth stage (D) the yellow discoloration of petals was initiated as well as the appearance of brown spots (Figure 1). Immediately after harvest, the petals were removed with a single-use blade and ground to a fine powder with liquid nitrogen. Total RNA was extracted according to the method of Bachem et al. 1998 [58] and quantified using the IMPLEN GmbH Nanophotometer/NanoPhotometerTM Pearl. RNA samples were stored at −80°C.

The isolated RNA was pooled for each floral stage and 400 μg of the pooled RNA (per stage) were diluted in 2.5 volumes of absolute ethanol and 0.1 volume of sodium acetate (pH 5.2) in DEPC-treated ddH2O. We performed de novo transcriptome sequencing in the four samples, using Illumina HiSeq™ 2000 (Beijing Genomics Institute - BGI, Hong Kong).

### Library preparation and sequencing

The RNA samples were treated with DNase I to ensure that are DNA-free. Subsequently the mRNAs were enriched by using the oligo (dT) magnetic beads and then fragmented into short pieces (about 200 bp). These fragments were used to synthesize the dsDNA using random hexamer primer for the first strand and DNA polymerase I for the second strand. Double stranded cDNA was purified with magnetic beads and subjected to end reparation and 3′ single adenylation. Sequencing adaptors were then ligated to the adenylated fragments, which were subsequently enriched by PCR amplification. The Agilent 2100 Bioanalyzer and ABI StepOnePlus Real-Time PCR System was used to qualify and quantify the sample library during the QC step. The library products were sequenced using Illumina HiSeqTM 2000.

### Data processing and de novo assembly

Raw reads that are obtained from the sequencer often contain low quality reads and/or adaptor sequences. Therefore, a preprocessing step of data cleaning was required to obtain the “clean reads” that would be used in the next steps of data analysis. This step included the adaptor removal as well as the application of a stringent filtering criterion to remove reads with >10% unknown bases and remove low quality reads, i.e. reads that comprise at least 50% low-quality bases. The reads that were obtained after the above mentioned filtering criteria were considered clean reads. These reads were used for the de novo assembly using Trinity [59] software that comprises three independent software modules, namely Inchworm, Chrysalis and Butterfly. Inchworm assembles the reads into longer transcripts in order to form contigs, Chrysalis groups the contigs into clusters that represent the transcriptional complexity of the gene and Butterfly reports the full-length transcripts for alternatively spliced isoforms. The output of Trinity are sequences called unigenes that can either form clusters in which the similarity among sequences is >70%, or unigenes that are unique and form the singletons. Lastly, all unigenes are aligned to protein databases including NR, Swiss-Prot, KEGG and COG (blastx, E-value <10^-5^) and the optimal result is used the sequence direction of unigenes. In the case of conflict among the results of the databases, the priority order of NR, Swiss-Prot, KEGG and COG is used. When the unigene is not aligned in any of the databases, ESTScan [60] was used to detect possible coding regions of the gene as well as identify the sequencing direction.

### Quantification of gene expression, differentially expressed genes and gene annotation

The gene expression levels are determined by calculating the RPKM (Reads Per kb per Million reads) values using the formula RPKM_A_ = 10^6^ * C_A_ (N * L_A_/10^3^)^-1^, where C_A_ is the number of reads that align uniquely to gene A, N is the total number of reads that align uniquely to all genes, and L_A_ is the number of bases of this gene. Fold changes between (pairs of) stages were computed as the ratio of RPKM values.

Identification of differentially expressed genes in a pair of conditions 1 and 2 is based on the p-value:


where N1 and N2 represent the total number of reads and x and y represent the number of transcripts for a particular gene A, in conditions 1 and 2, respectively. False Discovery Rate (FDR) is the ratio of false positive over true differentially expressed genes and is set to a threshold of FDR ≤ 0.001, while the absolute value of log_2_Ratio was set to 1 or larger. Differentially expressed genes are then carried out into GO functional analysis and KEGG Pathway analysis.

### Quantitative real-time PCR analysis

Expression analysis of the selected genes was assessed using quantitative RT- PCR. 2 μg of total RNA of each sample was converted into single stranded cDNA using TaKaRa PrimeScript Reverse Transcriptase (Cat. #2680A). The cDNA products were diluted 100-fold in DEPC ddH2O before used as templates. The reaction was performed on a Step One Plus Real-Time PCR System (Applied Biosystems, USA), using the Thermo Scientific Maxima SYBR Green/ROX qPCR Master Mix (2x) (Cat. #K0222). 20 μL of the reaction system contained: 10 μL of SYBR Green qPCR Master Mix (2×), 2 μL of each of the forward and reverse primers, 1 μL of water and 5 μL of cDNA template (1 ng/μL). The reaction conditions were performed as follows: 95°C for 10 min, followed by 40 cycles of 95°C for 15 sec, 60°C for 30 sec and 72°C for 30 sec. At least two biologically independent replicates were used for each sample. Average Ct values of actin and PP2A were used as internal reference genes for the normalization of the expression levels of the selected transcripts. Relative gene expression levels were calculated using the 2^-ΔΔCt^ [61]. The primer sequences used for qRT – PCR are listed in Additional file 5.

### Availability of supporting data

This Transcriptome Shotgun Assembly project has been deposited at DDBJ/EMBL/GenBank under the accession GAQP00000000.

## Electronic supplementary material

Additional file 1: **Summary of the read statistics of gardenia transcriptome generated by the Illumina platform.** (DOCX 11 KB)

Additional file 2: **Summary of quantification statistics in the four stages of gardenia transcriptome.** (DOCX 11 KB)

Additional file 3: **Statistics of the gardenia transcriptome against Nr database. (A)** E-value distribution. The E-value distribution of the top hits in the NCBI non-redundant (Nr) database indicates a strong homology (<1.0e-45) at 54.7% of the annotated unigenes, while the 45.3% showed a moderate homology (between 1.0e-5 and 1.0e-45) **(B)** Similarity distribution. The similarity distribution showed that the 25.4% of the annotated sequences had a similarity higher than 80% **(C)** Species distribution. On a species basis the majority (46.9%) of the sequences matched to Vitis vinifera [42] followed by Ricinus communis (14.0%). (TIFF 2 MB)

Additional file 4: **Statistics of the SSRs identified in gardenia transcriptome.** (DOCX 12 KB)

Additional file 5: **Primers used for the real-time quantitative PCR.** (XLSX 10 KB)
