# Supplementary material for: De novo transcriptome analysis of petal senescence in Gardenia jasminoides Ellis
Source: BMC Genomics. 2014 Jul 4;15(1):554. doi: 10.1186/1471-2164-15-554 (PMC4108791; doi:10.1186/1471-2164-15-554)
Supplement: Supplementary file 1 — Additional file 1: Summary of the read statistics of gardenia transcriptome generated by the Illumina platform. (DOCX 11 KB) [file 12864_2014_6265_MOESM1_ESM.docx]

|  | | | | | |
| --- | --- | --- | --- | --- | --- |
|  | **Raw Reads** | **Clean Reads** | **Contigs** | **Unigenes** | **Annotated** |
| **Transcriptome** | 55,092,396 | 50,335,672 | 102,263 | 57,503 | 39,459 |
