# Supplementary material for: De novo transcriptome analysis of petal senescence in Gardenia jasminoides Ellis
Source: BMC Genomics. 2014 Jul 4;15(1):554. doi: 10.1186/1471-2164-15-554 (PMC4108791; doi:10.1186/1471-2164-15-554)
Supplement: Supplementary file 2 — Additional file 2: Summary of quantification statistics in the four stages of gardenia transcriptome. (DOCX 11 KB) [file 12864_2014_6265_MOESM2_ESM.docx]

|  | | | |
| --- | --- | --- | --- |
| **Sample ID** | **Total reads** | **Reads with unique match** | **Unigenes** |
| **A** | 13,518,781 | 8,566,413 (63.37%) | 46,547 |
| **B** | 13,050,688 | 8,372,935 (64.16%) | 45,886 |
| **C** | 13,691,764 | 8,614,148 (62.91%) | 45,477 |
| **D** | 12,818,378 | 8,085,437 (63.08%) | 44,912 |
