# Supplementary figures and images for: De novo transcriptome analysis of petal senescence in Gardenia jasminoides Ellis
Source: BMC Genomics. 2014 Jul 4;15(1):554. doi: 10.1186/1471-2164-15-554 (PMC4108791; doi:10.1186/1471-2164-15-554)

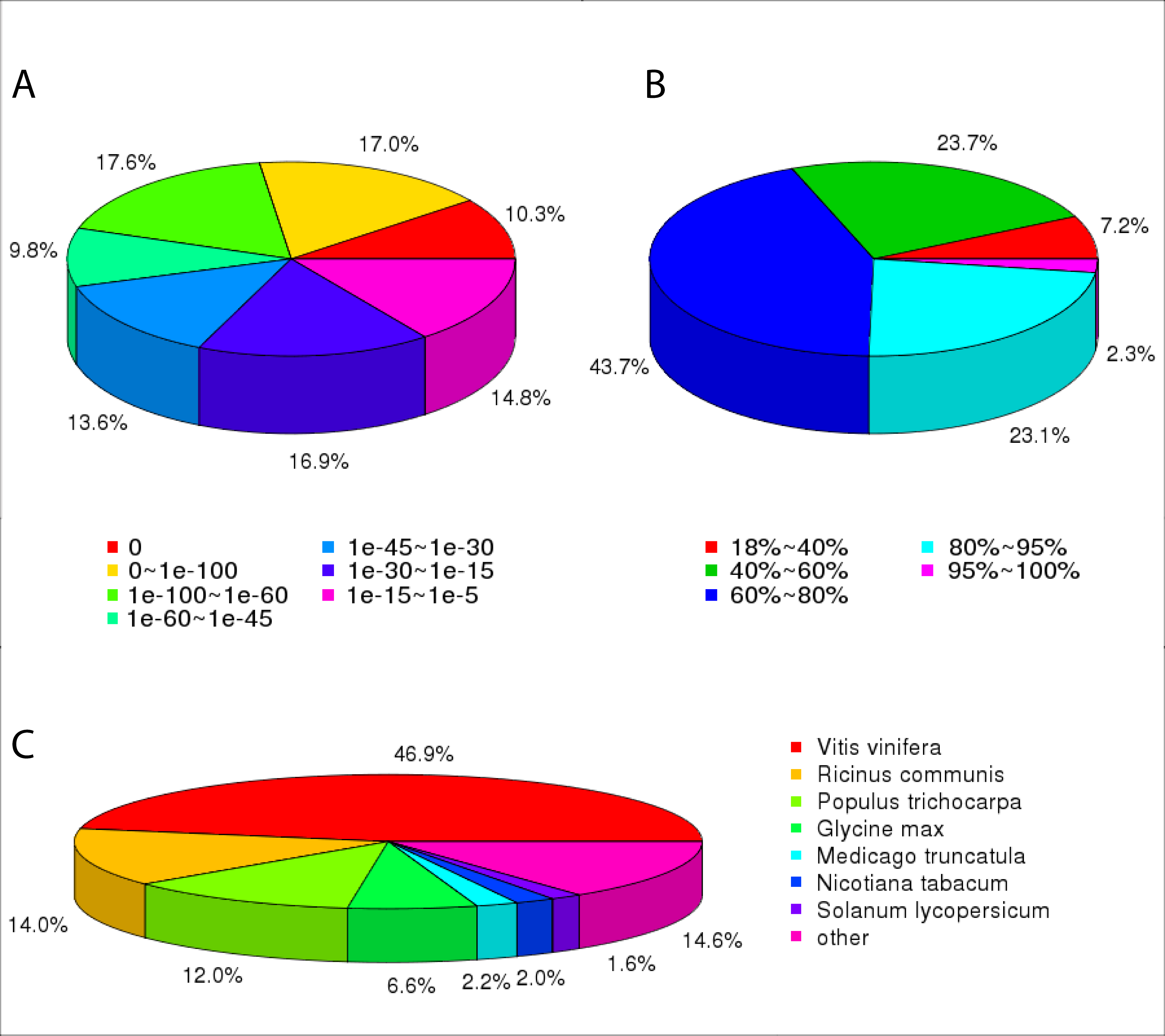

Supplement: Supplementary file 3 — Additional file 3: Statistics of the gardenia transcriptome against Nr database. (A) E-value distribution. The E-value distribution of the top hits in the NCBI non-redundant (Nr) database indicates a strong homology (<1.0e-45) at 54.7% of the annotated unigenes, while the 45.3% showed a moderate homology (between 1.0e-5 and 1.0e-45) (B) Similarity distribution. The similarity distribution showed that the 25.4% of the annotated sequences had a similarity higher than 80% (C) Species distribution. On a species basis the majority (46.9%) of the sequences matched to Vitis vinifera [42] followed by Ricinus communis (14.0%). (TIFF 2 MB) [file 12864_2014_6265_MOESM3_ESM.tiff]
