# Supplementary material for: De novo transcriptome analysis of petal senescence in Gardenia jasminoides Ellis
Source: BMC Genomics. 2014 Jul 4;15(1):554. doi: 10.1186/1471-2164-15-554 (PMC4108791; doi:10.1186/1471-2164-15-554)
Supplement: Supplementary file 4 — Additional file 4: Statistics of the SSRs identified in gardenia transcriptome. (DOCX 12 KB) [file 12864_2014_6265_MOESM4_ESM.docx]

|  | |
| --- | --- |
| **Parameter** | **Value** |
|  |  |
| **SSR Identification** |  |
| Total number of sequences examined | 57,503 |
| Total size of examined sequences (bp) | 45,780,886 |
| Total number of identified SSR’s | 9,549 |
| Number of SSR containing sequences | 7,641 (13.3%) |
| Number of sequences containing more than one SSR | 1,398 (18.3%) |
|  |  |
| **Distribution to different repeat type classes** |  |
| Mononucleotide | 3,129 (32.7%) |
| Dinucleotide | 2,853 (29.8%) |
| Trinucleotide | 2,937 (30.7%) |
| Tetranucleotide | 230 (2.4%) |
| Pentanucleotide | 209 (2.2%) |
| Hexanucleotide | 191 (2.0%) |
